# Fluorescence signatures of SARS-CoV-2 spike S1 proteins and a human ACE-2: excitation-emission maps and fluorescence lifetimes

**DOI:** 10.1117/1.JBO.27.5.050501

**Published:** 2022-05-28

**Authors:** Jonas Grzesiak, Lea Fellner, Karin Grünewald, Christoph Kölbl, Arne Walter, Reinhold Horlacher, Frank Duschek

**Affiliations:** a German Aerospace Center (DLR), Institute of Technical Physics, Hardthausen, Germany; b Trenzyme GmbH, Konstanz, Germany

**Keywords:** excitation-emission maps, fluorescence decay times, fluorescence, Severe acute respiratory syndrome coronavirus 2, human angiotensin-converting enzyme 2

## Abstract

**Significance:**

Fast and reliable detection of infectious SARS-CoV-2 virus loads is an important issue. Fluorescence spectroscopy is a sensitive tool to do so in clean environments. This presumes a comprehensive knowledge of fluorescence data.

**Aim:**

We aim at providing fully featured information on wavelength and time-dependent data of the fluorescence of the SARS-CoV-2 spike protein S1 subunit, its receptor-binding domain (RBD), and the human angiotensin-converting enzyme 2, especially with respect to possible optical detection schemes.

**Approach:**

Spectrally resolved excitation-emission maps of the involved proteins and measurements of fluorescence lifetimes were recorded for excitations from 220 to 295 nm. The fluorescence decay times were extracted by using a biexponential kinetic approach. The binding process in the SARS-CoV-2 RBD was likewise examined for spectroscopic changes.

**Results:**

Distinct spectral features for each protein are pointed out in relevant spectra extracted from the excitation-emission maps. We also identify minor spectroscopic changes under the binding process. The decay times in the biexponential model are found to be (2.0±0.1) ns and (8.6±1.4)  ns.

**Conclusions:**

Specific material data serve as an important background information for the design of optical detection and testing methods for SARS-CoV-2 loaded media.

## 1 Introduction

The global spread of the severe acute respiratory syndrome coronavirus 2 (SARS-CoV-2) virus, which started in early 2020^1^ and which was declared a pandemic by the World Health Organization in March 2020,^2^ has promoted intensive research on human coronaviruses,^3^ which are a global public health threat.^4^ A major tool in monitoring and controlling the spread of the viruses is fast, accurate, and sensitive detection of the virus or the infection.^5^ In the case of SARS-CoV-2, a significant number of research articles on sampling techniques, nanobiosensor technologies, and antigen testing have been published and are reviewed, e.g., in Refs. 6–9. An overview on photonic approaches for detecting virus loads and infections by optical means is given in Ref. 10. The authors summarize that the diversity of viruses and the unique microbiomes in humans are still a challenge for spectroscopic techniques such as Fourier-transform infrared spectroscopy, Raman, and fluorescence spectroscopy, in terms of identifying one specific single virus in their spectra. Among those methods, laser-induced fluorescence (LIF) is a widely used tool to investigate proteins.^11^^–^^14^ Elastic light scattering and fluorescence have been used to observe virus particles and virus-like particles.^15^^–^^17^ Compared to Raman spectroscopy, the LIF technique yields higher signal intensities and has been applied to detect and classify viruses with excitation in the near-ultraviolet (near-UV).^18^^,^^19^ As pointed out in Ref. 20, additional orthogonal information on fluorescence characteristics can be retrieved not only from two-dimensional, spectral signatures (excitation-emission maps) but also from fluorescence decay times. Such distinct features are of high importance for a reliable sample classification.^21^

In this work, high-resolution fluorescence data of the proteins involved in the SARS-CoV-2 binding process, namely the S1 part of the spike protein of SARS-CoV-2, the receptor-binding domain thereof, and human angiotensin-converting enzyme 2 (hACE2) are reported as well as results on their fluorescence decay times. Spectral data and fluorescence decay times of the investigated proteins are compared and discussed as candidates for classification features.

## 2 Experimental Setup

The proteins used for this study are SARS-CoV-2 S1 RBD (receptor binding domain) (S1 RBD domain with His-Tag, M=27.5  kDa), SARS-CoV-2 S1 (S1 domain with His-tag, 77.1 kDa), and hACE2 (ECD domain, tag-free processes M=80  kDa). The proteins were produced by trenzyme GmbH using a transient production system in HEK293 suspension cells followed by purification either via the encoded His-Tag by IMAC (RBD and S1 protein) or by ion-exchange chromatography (hACE2). After purification, the buffer of the purified proteins was exchanged to Dulbecco’s phosphate-buffered saline (DPBS), (pH=7.1−7.5, Sigma-Aldrich) and the proteins were analyzed. The final concentrations of the solutions were 22.6  μg/ml in the case of the hACE2, 7.8  μg/ml in the RBD case, and 22.2  μg/ml for the full-length S1 protein. This ensures comparable molar concentrations of the proteins for the analysis.

The samples were investigated in UV-transparent cuvettes (fused silica glass, Hellma 117F), with a light travel path of 10 mm in the spectrometer.

An FS5 fluorescence spectrometer from Edinburgh Instruments was used to obtain full fluorescence signatures of the proteins. The spectrometer uses a 90 deg setup and a variable wavelength excitation. Additionally, it allows for time-correlated single-photon measurements of the samples. The fluorescence maps contain data for excitation wavelengths ranging from 220 to 300 nm and emissions from 230 to 500 nm. Time-dependent fluorescence signals have been recorded at excitation at 260 nm (wavelength of the short pulse light emitting diode module), with 0.9 ns excitation pulse duration and the respective optimal emission wavelengths.

## 3 Results and Discussion

### 3.1 LIF Spectral Signatures of the hACE2 Enzyme and the S1 Protein

**Fig. 1:**
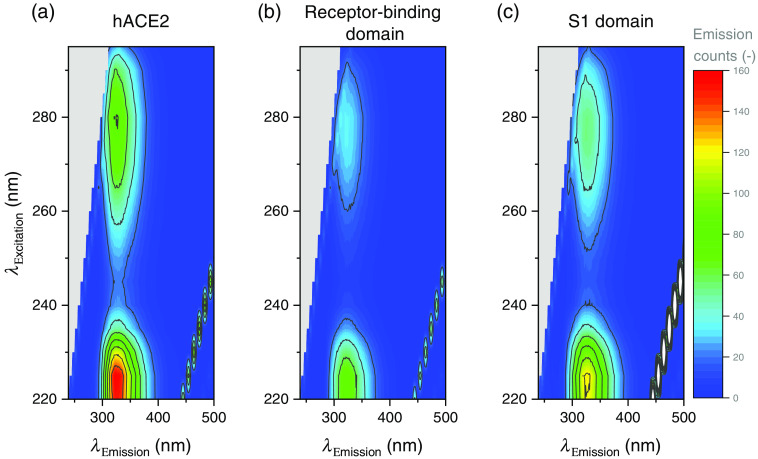
Excitation-emission fluorescence map for (a) hACE2, (b) the receptor-binding domain, and (c) SARS-CoV-2 S1. Data are available; see Code, Data, and Materials Availability.

As an overview, the emission maps of the SARS-CoV-2 S1 spike protein, RBD the thereof, and the hACE2 are shown in Fig. 1. The resolution is 5 nm for the excitation wavelength axis and 3 nm for the emission wavelength axis. The diagonal trace of peaks, at $+3430\;{\mathrm{cm}}^{-1}$ from the excitation wavelengths $\lambda_{\mathrm{ex}}$ are assigned to be the Raman signals of $H_{2}O$. The narrow peaks in the lower right corner of the graphs are assigned to be the second-order diffraction maximum. Each map consists of two major maxima: the first one located at an excitation wavelength $\lambda_{\mathrm{ex}}=220\;\mathrm{nm}$ with maximum emission at $\lambda_{\mathrm{em}}=325\;\mathrm{nm}$ for S1 and hACE2 and at $\lambda_{\mathrm{em}}=320\;\mathrm{nm}$ for the receptor-binding domain. The second major maximum appears at an excitation wavelength of $\lambda_{\mathrm{ex}}=280\;\mathrm{nm}$ showing a maximum emission at $\lambda_{\mathrm{em}}=325\;\mathrm{nm}$ for all three proteins.

**Fig. 2:**
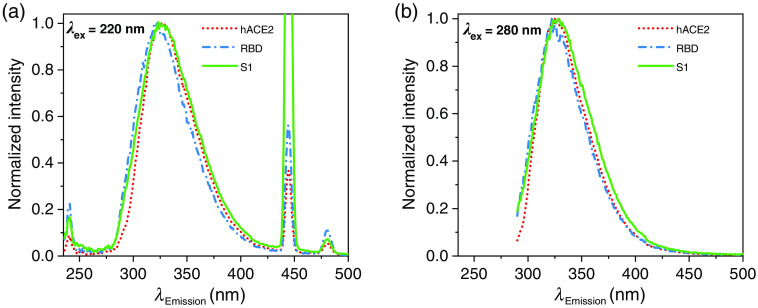
Detailed, normalized fluorescence signals of the three proteins at (a) $\lambda_{\mathrm{ex}}=220\;\mathrm{nm}$ and (b) $\lambda_{\mathrm{ex}}=280\;\mathrm{nm}$.

**Fig. 3:**
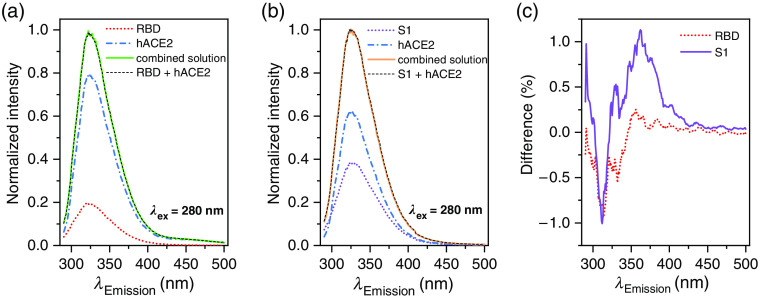
Fluorescence spectra of bound protein complexes and their single contributions: (a) receptor-binding domain of the S1 protein, (b) the full S1 protein, and (c) the differences between the added single contributions and the bound complex for the RBD and the S1 case.

Figure 2 displays the normalized fluorescence response of the three samples in detail at excitation wavelengths λ=220  nm and $\lambda_{\mathrm{ex}}=280\;\mathrm{nm}$. For clearness, the emission spectra obtained at $\lambda_{\mathrm{ex}}=280\;\mathrm{nm}$ have been adjusted by the background signal from a pure PBS sample. The signals at $\lambda_{\mathrm{ex}}=220\;\mathrm{nm}$ clearly show the Raman signal of the solvent as well as the second-order diffraction maximum. The shown signals are single measurements with a noise level of less than 1% of the maximum peak intensity for the $\lambda_{\mathrm{ex}}=220\;\mathrm{nm}$ case and less than 5% for the $\lambda_{\mathrm{ex}}=280\;\mathrm{nm}$ case due to lower signal intensities. The intensities of the respective signals are indicated by the Raman signal intensities and are more explicit in Fig. 3. The fluorescence peaks are located between 290 and 410 nm, which indicates that the excitation light mainly probes tryptophan and tyrosine sites inside the proteins.^11^ At both respective excitation wavelengths, the three proteins show significantly different fluorescence responses. This is manifested in differently shaped peaks, which in turn give full width half maximum (FWHM) values of the peaks from 55 nm for hACE2 and the receptor-binding domain to 60 nm for the S1 protein, both at an excitation wavelength of $\lambda_{\mathrm{ex}}=280\;\mathrm{nm}$. The same pattern can be seen at $\lambda_{\mathrm{ex}}=220\;\mathrm{nm}$, with slightly broader values for FWHM: 58 nm for hACE2 and RBD and 63 nm for the S1 protein. These slightly broader spectra may emerge from an excited hot (i.e., vibrationally excited) state with a broad emission spectrum due to the lower excitation wavelength.

Besides the pure protein signals, the bound complex of the virus with its receptor is of interest for a possible virus detection. To obtain a protein–enzyme complex solution we combined both the solution of the S1 full-length protein and the RBD protein solution with the hACE2 solution: 1 ml of each solution with the respective concentrations noted above to ensure equimolarity of both substances in the combined solution. Immediately following the combination, the mixture was stirred to ensure a fully mixed solution. From the receptor binding affinity of about 1 to 40 nM^22^ and the concentration of the S1 protein and the RBD protein analytes of about $3.0\cdot 10^{-7}\;\frac{\mathrm{Mol}}{l}$ one obtains a half-life of the binding process in the range of τ≈3 to 35 s. Thus, the full binding of all hACE2 receptors to the virus proteins in our solution is ensured within 5 min, when the spectra were taken. Figures 3(a) and 3(b) show the fluorescence results of the bound complexes at an excitation wavelength of 280 nm. The signals have been adjusted by the background signal from a pure PBS sample and for simplicity, the normalized signals are shown. For comparison, the single contributions are shown too. We compared the signal of the bound complexes with the sum of the signals of the single contributions (black dashed lines in the spectra). The difference spectra between the summed signals and the bound complexes are shown in Fig. 3(c). Here, the significant change of about −1% of the signal at 312 nm is due to differences in the $H_{2}O$ Raman signal. The average noise is about 0.17% and from the spectral resolution, we expect the relevant feature to be at 350 to 400 nm: a slight change in the slopes of the peak is found, indicating a possible conformation change under the binding process, especially in the S1 case.

### 3.2 Fluorescence Decay Times

**Fig. 4:**
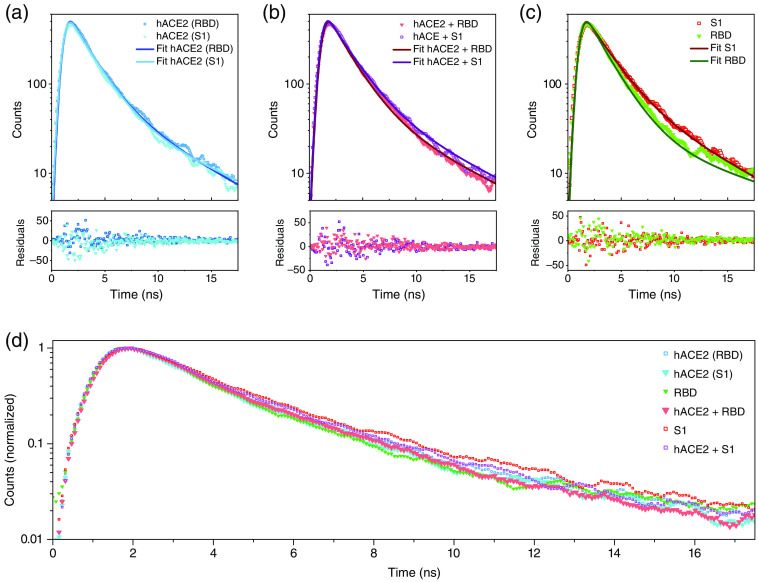
Time correlated single photon counting signals for the different solutions and their respective double exponential decay fit and the residuals: (a) for the hACE protein, (b) for the combination of hACE with RBD and S1, and (c) for the spike protein parts S1 and RBD. (d) A summary of all measured traces for comparison.

Additionally, the fluorescence decay of the base solutions of the hACE2, the RBD protein, and the S1 protein as well as of their bound complex solutions were measured at an excitation of 260 nm as a time-correlated single-photon counting signal, shown in Fig. 4. The fluorescence signals can be described by a double exponential decay. In contrast to other related multiexponential descriptions such as the identification of the two lifetimes in tryptophan^23^ or the detection of different fluorescent components,^24^ found lifetimes are not necessarily dedicated to specific physical processes of the protein or its constituents, but of descriptive nature. However, structural rearrangements of the proteins during the binding process may lead to changes in the observed decay constants. Here, the fits were performed as global fits, where all signals are taken into account at once and the fit for the bound complexes is fitted as a sum of the single contributions. The fit function is a Gaussian-shaped excitation function convolved with a bi-exponential decay to describe the fluorescence decay. The fit routine was performed with Origin Pro 2021 using a Levenberg–Marquardt iteration and restricting the parameters only to be non-negative. Results of the fits are summarized in Table 1, the error noted is the error due to the fit routine. For both global fits, the coefficient of determination is $R^{2}=0.99$. The fit analysis for the decays yields that the two-time constants in the exponential decay, $\tau_{1}$ and $\tau_{2}$, are only slightly different for all proteins and bound protein complexes. The recorded signals consist of a fast and slow decay with $\tau_{1}\approx (2.0\pm 0.1)$ and $\tau_{2}\approx (8.6\pm 1.4)\;\mathrm{ns}$ on average for all proteins. Since the proteins contain multiple sites of tryptophan and tyrosine and the fluorescence is a mixture of the single fluorescing sites, it is difficult to connect the two lifetimes with specific single fluorophores. The average lifetimes for all proteins vary also only slightly and are on average $\tau_{\mathrm{avg}}\approx (5.9\pm 0.9)\;\mathrm{ns}$.

**Table 1:** Results of the double exponential fit to the different protein time-correlated single-photon signals at an excitation wavelength of 260 nm. The errors are the standard errors obtained by the fit routine.

	$\tau_{1}$ (ns)	$\tau_{2}$ (ns)	$\tau_{\mathrm{avg}}$ (ns)
hACE2	1.9±0.1	7.5±1.4	5.5±1.0
RBD	1.9±0.1	11.4±3.8	6.2±2.2
S1	2.1±0.1	6.9±1.5	6.2±1.3

## 4 Summary

We have presented fluorescence data for the S1 spike protein of the SARS-CoV-2, its receptor-binding domain, and its most important receptor in human organisms, hACE2. The spectra show distinguishable features such as slight, but clear shifts in the shapes of the LIF signatures for all proteins, e.g., manifested in different FWHMs of the respective fluorescence peaks. Additionally, the signals vary with the excitation wavelength and show for example a clear blueshift of about 5 nm for the receptor-binding domain at $\lambda_{\mathrm{ex}}=220\;\mathrm{nm}$ in contrast to $\lambda_{\mathrm{ex}}=280\;\mathrm{nm}$. Assuming a biexponential fluorescence lifetime model, the fluorescence decay times have been determined to be $\tau_{1}\approx (2.0\pm 0.1)$ and $\tau_{2}\approx (8.6\pm 1.4)\;\mathrm{ns}$ on average for all proteins. The average lifetimes are determined to be $\tau_{\mathrm{avg}}\approx (5.9\pm 0.9)\;\mathrm{ns}$. For further insight, the limitations are the signal-to-noise ratios and the maximum possible integration times in order to avoid photolytic decomposition of the proteins in the UV. The data are publicly available for external R&D as described in Code, Data, and Materials Availability.

## Code, Data, and Materials Availability

Measurement data is available as follows:

Data Combined Spectra, S1

Data Combined Spectra, RBD

Emission Map, hACE2

Emission Map, S1

Emission Map, RBD

Spectrum, PBS

TCSPC S1

TCSPC RBD

Fit Formula and PBS Spectrum.
