# Supplementary material for: Fluorescence signatures of SARS-CoV-2 spike S1 proteins and a human ACE-2: excitation-emission maps and fluorescence lifetimes
Source: J Biomed Opt. 2022 May 28;27(5):050501. doi: 10.1117/1.JBO.27.5.050501 (PMC9142794; doi:10.1117/1.JBO.27.5.050501)
Supplement: Supplementary file 9 [file JBO_027_050501_SD009.pdf]

Supplementary material for the article

'Fluorescence signatures of SARS-CoV-2 spike S1 proteins and an human ACE-2: excitation-emission maps and fluorescence lifetimes'

The formula used for the biexponential fit is a convolution of a gaussian shaped instrument function:

$$f(x) = \frac{a}{\sqrt{2\pi}\omega} e^{\frac{-(x-t_0)^2}{2\omega^2}}$$

with a width ω and the center t_0, and a bi-exponential decay:

$$g(x) = \left(\alpha_1 e^{-\tau_1(x)} + \alpha_2 e^{-\tau_2(x)}\right)$$

with its amplitudes α_i and the decay constants τ_i. The convolution is then given by

$$\begin{aligned} I(t) &= \int_{-\infty}^{t} f(x) * g(t-x)dx \\ &= \int_{-\infty}^{t} \frac{a}{\sqrt{2\pi}\omega} e^{\frac{-(x-t_0)^2}{2\omega^2}} \cdot \left(\alpha_1 e^{-\tau_1(t-x)} + \alpha_2 e^{-\tau_2(t-x)}\right) dx \\ &= a \cdot \alpha_1 \cdot e^{\frac{1}{2}\tau_1(\tau_1 \cdot \omega^2 + 2t_0 - 2t)} \cdot \left[1 - erf\left(\frac{t_0 + \tau_1\omega^2 - t}{\sqrt{2}\omega}\right)\right] \\ &+ a \cdot \alpha_2 \cdot e^{\frac{1}{2}\tau_2(\tau_2 \cdot \omega^2 + 2t_0 - 2t)} \cdot \left[1 - erf\left(\frac{t_0 + \tau_2\omega^2 - t}{\sqrt{2}\omega}\right)\right]. \end{aligned}$$

The PBS emission spectrum of PBS at λ_{ex} =280 nm is shown in figure 1. Additionally, the emission spectrum for the S1 protein in comparison to the the PBS spectrum is shown.

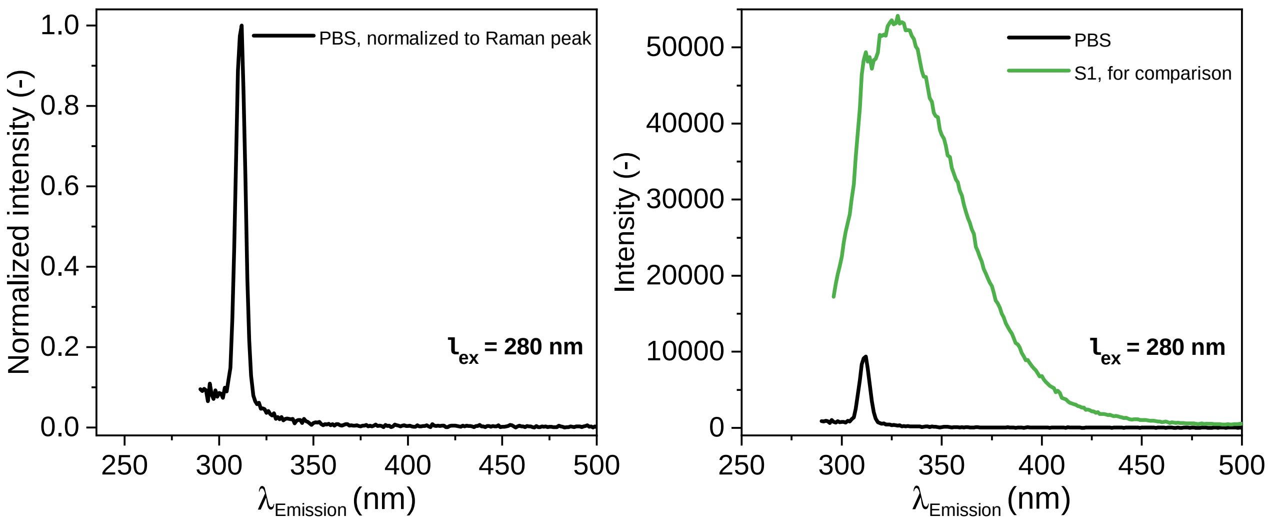

Figure 1: Emission spectrum of PBS at λ_{ex} =280 nm (left), emission spectra of PBS and S1 at λ_{ex} =280 nm (right)
